# IT support in emergency remote teaching in response to COVID-19

**DOI:** 10.3205/zma001412

**Published:** 2021-01-28

**Authors:** Christian Bruns, Tim Herrmann, Martin Böckmann-Barthel, Hermann-Josef Rothkötter, Johannes Bernarding, Markus Plaumann

**Affiliations:** 1 Otto von Guericke University Magdeburg, Institute of Biometry and Medical Informatics, Magdeburg, Germany; 2 Otto von Guericke University Magdeburg, Data Integration Center, Magdeburg, Germany; 3 Otto von Guericke University Magdeburg, Department of Experimental Audiology, Magdeburg, Germany; 4 Otto von Guericke University Magdeburg, Institute of Anatomy, Magdeburg, Germany

**Keywords:** emergency remote teaching, virtual rooms, IT support, medical education, COVID-19

## Abstract

**Background: **The COVID-19 pandemic hit the German education system unexpectedly and forced its universities to shift to Emergency Remote Teaching (ERT). The Data Integration Center (DIC) of the University Hospital Magdeburg and the Institute of Biometry and Medical Informatics (IBMI) has developed a concept based on existing structures that can be quickly implemented and used by the Medical Faculty at Otto von Guericke University. This manuscript focuses on the IT support for lecturers, which allows them to concentrate on teaching their lessons, although the authors are aware that this is only a small part of the entire subject. Additionally, there is a great awareness that ERT can never replace well-structured in-person classes.

**Concept: **The key feature of the concept uses the well-working management system for all physical rooms of the university by designing a virtual video conference room for every physical room. This allows high interactivity for lectures and seminars while applying proven teaching methods. Additionally, a collaboration software system to document all lessons learned and a technical support team have been available for the teaching staff. Courses with a hands-on approach require more personal interaction than lectures. Therefore, the issues of practical trainings have not been solved with this concept, but been tackled by using questionnaires and minimizing contacts during attestations.

**Applied IT tools: **The concept’s requirements were met by *Zoom Meetings, Confluence, HIS/LSF* and *Moodle*.

**Discussion and Conclusion: **The concept helped the lecturers to provide high-quality teaching for students at universities. Additionally, it allows for a dynamic response to new needs and problems. The concept will be reviewed as part of a higher *Universal Design for Learning* concept and may support lecturers in the following semesters in hybrid meetings with real and virtual attendees.

## Zusammenfassung

**Hintergrund: **Die Covid-19-Pandemie traf das deutsche Bildungssystem unerwartet und zwang dessen Universitäten, auf „Emergency Remote Teaching“ (ERT) umzustellen. Das Datenintegrationszentrum (DIZ) des Universitätsklinikums Magdeburg und das Institut für Biometrie und Medizinische Informatik (IBMI) haben auf Grundlage bestehender Strukturen ein Konzept entwickelt, das von der Medizinischen Fakultät der Otto-von-Guericke-Universität schnell umgesetzt und genutzt werden konnte. Im Mittelpunkt dieses Manuskripts steht die IT-Unterstützung der Dozierenden, die es diesen ermöglicht, sich auf die Lehre zu konzentrieren, wobei den Autoren bewusst ist, dass dies nur einen kleinen Teil des gesamten Problems abdeckt. Außerdem ist dieses Manuskript unter dem Bewusstsein verfasst, dass die ERT einen gut strukturierten Präsenzunterricht niemals ersetzen kann.

**Konzept: **Das Hauptmerkmal des Konzepts ist die weitere Nutzung des bereits gut funktionierenden Raumverwaltungssystems für alle existierenden Räume der Universität mithilfe der Einrichtung eines virtuellen Videokonferenzraums für jeden physischen Raum. Dies ermöglicht eine hohe Interaktivität für Vorlesungen und Seminare bei weiterer Verwendung bewährter und gewohnter Lehrmethoden. Zusätzlich wurden ein Kollaborationssoftwaresystem zur Dokumentation aller gewonnenen Erkenntnisse und typischer Fehler und ein technisches Support-Team für das Lehrpersonal zur Verfügung gestellt. Da Kurse mit einem praxisorientierten Ansatz mehr persönliche Interaktion als Vorlesungen benötigen, wurden die Anforderungen der Praktika nicht vollständig mit diesem Konzept gelöst, aber durch die Verwendung von Fragebögen und der Minimierung von Kontakten während der Testate angegangen.

**Angewandte IT-Tools: **Die Anforderungen des Konzepts wurden durch* Zoom Meetings, Confluence, HIS/LSF* und *Moodle* gelöst.

**Diskussion und Schlussfolgerung: **Das Konzept half den Dozierenden den Studierenden an der Hochschule eine hochqualitative Lehre anzubieten. Darüber hinaus ermöglicht es eine dynamische Reaktion auf neue Bedürfnisse und Probleme. Das Konzept wird im Rahmen eines „Universal Design for Learning“-Konzepts überprüft und kann die Dozierenden in den folgenden Semestern bei hybriden Veranstaltungen mit realen und virtuellen Teilnehmern unterstützen.

## Introduction

The COVID-19 pandemic arrived in Germany just before the start of the summer semester 2020 and caused dramatic change in traditional teaching at German universities [1], which had mostly been based on in-person classes. This report focuses on a concept which enabled the Medical Faculty of the Otto von Guericke University Magdeburg to provide as many courses as possible using IT resources. Building on an existing minimal e-learning structure and experiences with virtual conference tools, the main goal was to provide the lecturers in cooperation with the Studies Office with an IT infrastructure they could use almost effortlessly to provide the students with their existing teaching materials. Therefore, the authors decided to talk to the lecturers at a meta-level about their main needs and IT skills before developing a fast and user-friendly generic IT solution for everyone.

The authors want to emphasize that Emergency Remote Teaching (ERT) [2] as applied in this case can never replace a well-thought-out step-by-step process adapting each part of the teaching chain [3]. Nonetheless, the COVID-19 crisis afforded a new insight into different IT solutions that provide the technical basis for teaching a large part of the required materials instead of shutting down the university completely to make the required methodological adaptations. The different requirements and the corresponding IT solutions are described below.

## Administration

To put the concept decribed above into practice, the authors decided to use the university room bookings system for all existing physical rooms (LSF; [http://his.de]) to administrate the virtual rooms as well: a video conference room, which is permanently open and secured by a uniform password, was assigned to every physical room. Each physical room in the database is now directly linked to its corresponding video conference room. This has led to only a minimal change in the workflow and the workload of the University room bookings team. The concept was demonstrated to the lecturers online, which was recorded for future use. 

For additional technical support of the Medical Faculty, the Data Integration Center (DIC) of the University Hospital Magdeburg and the Institute of Biometry and Medical Informatics (IBMI) provided and trained a small four-person team that documented all lessons learned and recorded tutorial videos to provide and increase sustainable knowledge within the teaching staff using the collaboration software.

## Lectures/seminars

Most of the modern lecture strategies require at least a minimal interaction between lecturers and students. Therefore, this video conference solution permits showing the presentation of the lecturers and providing them with rights to unmute students for questions or comments. Another advantage is the possibility of using the same solution for lectures and seminars with high interactivity. However, the high number of students in medical courses requires licenses for at least 250 participants that might attend a lecture at the same time. Some lecturers are used to interacting more intensively with their students. They like to keep them alert and attentive by asking questions and involving them in possible medical decisions. For this, a standardized poll with generic answers (a, b, c, d, ...) was developed with regard to the content of the slides shown by the lecturers. To enable students to work in smaller groups, the virtual room can be split up by the lecturer.

### Practical trainings

Practical trainings and medical demonstrations can hardly be completely virtualized. One concept is to work in small groups to comply with social distancing rules. Additionally, in certain cases initial attestations via video had already reduced the number of contacts. As an alternative solution, experiments can be performed with various typical parameters and be recorded by a training supervisor. Afterwards, the students decide on the best steps to proceed with the experiment and obtain results by answering a questionnaire. Then they continue their work and log the results. Some of these experiments have been transformed into a step-by-step online course providing experimental data that relies on the individual decisions. 

#### IT tools

Software solutions matching the requirements presented above were *Zoom Meetings*, a videoconferencing tool, *Confluence*, a collaboration software from Atlassian, and Moodle, an already established learning platform for questionnaires of the practical trainings.

## Discussion

**Figure 1:**
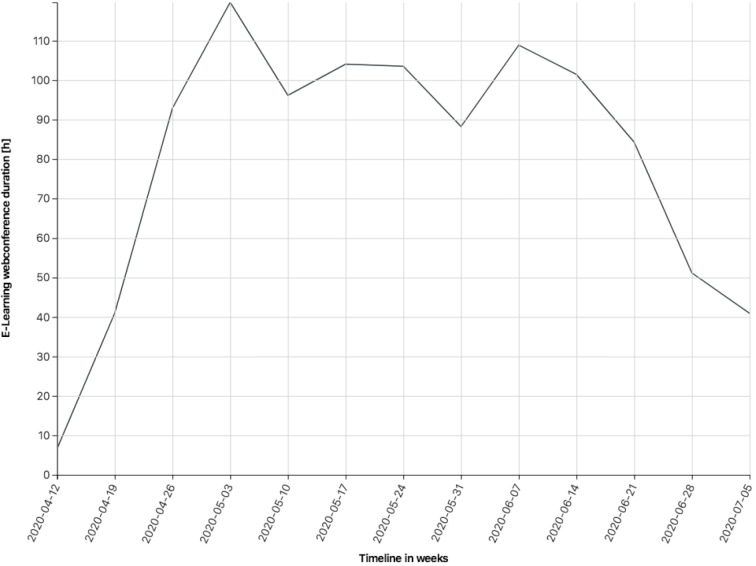
The usage statistics in hours as sum per week of 19 selected permanent (24h/7d) web conference meeting rooms for e-learning.

The concept has been more successful than expected (considering that this was ERT) not least because of the high motivation (also shown in figure 1 (Fig. 1) by the usage statistics from selected virtual rooms) and creativity of the lecturers themselves, optimizing and developing concepts for their own applications. Nonetheless, there were different typical misunderstandings in the beginning, i.e. lecturers were worried about overbooking the virtual rooms – just like they might worry about overbooking physical rooms. Hence, the team added some more rooms with an open calendar, which can be booked spontaneously and easily.

A first short evaluation by 24 lecturers against the background of ERT appreciated our intuitive solution. Lecturers noticed a higher attendance rate and asked for more customizable rooms for individual teaching in the future. Criticism was mainly voiced about the information provided beforehand to the lecturers. Hence, more information and video clips were provided in the collaboration software. Another criticism was the poor technical equipment, which has been neglected at our hospital just like at other German institutions in the past years [4]. The good students’ marks in exams and their very positive feedback were surprising. Noteworthy, the students supported each other a lot, leading to lower central technical support from University institutions.

The support level decreased massively as time went by, from around 8 hours per week in the beginning to just some calls at the end of the semester. In general, the IT support mainly focused on the videoconferencing tool.

The focus of this short manuscript is on IT support for lecturers. When physical attendance was necessary, i.e. during patient contacts or exams, the IT team was less able to provide support. Several other aspects are not covered in this report. Among them are privacy laws, students’ perspectives, the sudden change of didactic methods and a higher workload in clinical daily life due to the COVID-19 pandemic [4]. An obvious aspect of security should however be noted: in the summer semester, no non-authorized attendees were documented. Once, a password was leaked to an external person. The problem was solved within a few hours by changing the password. 

In conclusion, ERT cannot substitute personal contact, but it can prevent a complete shutdown of education institutions. Additionally, this “enforced experiment” has stimulated online education to supplement traditional teaching in the future. 

## Outlook

In the near future, the authors want to collect some more feedback to create a *Universal Design for Learning* concept [2] which includes IT infrastructure in preparation of future emergency teaching events. The authors are convinced that hybrid methods are possible with some of the participants attending in person while others participate virtually.

## Competing interests

The authors declare that they have no competing interests.
